# The relationship between the number of COVID-19 vaccines and infection with Omicron ACE2 inhibition at 18-months post initial vaccination in an adult cohort of Canadian paramedics

**DOI:** 10.1099/acmi.0.000725.v3

**Published:** 2023-11-28

**Authors:** Justin Yap, Iryna Kayda, Michael Asamoah-Boaheng, Scott Haig, Tracy Kirkham, Sheldon Cheskes, Paul Demers, David Goldfarb, Brian E. Grunau

**Affiliations:** ^1^​ British Columbia Resuscitation Research Collaborative, Vancouver, British Columbia, Canada; ^2^​ Faculty of Science, University of British Columbia, Vancouver, British Columbia, Canada; ^3^​ Experimental Medicine Graduate Program, Faculty of Medicine, University of British Columbia, Vancouver, British Columbia, Canada; ^4^​ Centre for Advancing Health Outcomes, St. Paul’s Hospital, Vancouver, British Columbia, Canada; ^5^​ Department of Emergency Medicine, University of British Columbia, Vancouver, British Columbia, Canada; ^6^​ BC Emergency Health Services, British Columbia, Canada; ^7^​ Dalla Lana School of Public Health, University of Toronto, Toronto, Ontario, Canada; ^8^​ The Occupational Cancer Research Centre, Ontario Health, Ontario, Canada; ^9^​ Department of Family and Community Medicine, Division of Emergency Medicine, University of Toronto, Toronto, Ontario, Canada; ^10^​ Department of Pathology and Laboratory Medicine, University of British Columbia, Vancouver, British Columbia, Canada

**Keywords:** COVID-19, omicron, SARS-CoV-2, spike, vaccination

## Abstract

The coronavirus disease 2019 (COVID-19) pandemic, caused by the SARS-CoV-2 virus, has rapidly evolved since late 2019, due to highly transmissible Omicron variants. While most Canadian paramedics have received COVID-19 vaccination, the optimal ongoing vaccination strategy is unclear. We investigated neutralizing antibody (NtAb) response against wild-type (WT) Wuhan Hu-1 and Omicron BA.4/5 lineages based on the number of doses and past SARS-CoV-2 infection, at 18 months post-initial vaccination (with a Wuhan Hu-1 platform mRNA vaccine [BNT162b2 or mRNA-1273]). Demographic information, previous COVID-19 vaccination, infection history, and blood samples were collected from paramedics 18 months post-initial mRNA COVID-19 vaccine dose. Outcome measures were ACE2 percent inhibition against Omicron BA.4/5 and WT antigens. We compared outcomes based on number of vaccine doses (two vs. three) and previous SARS-CoV-2 infection status, using the Mann-Whitney U test. Of 657 participants, the median age was 40 years (IQR 33–50) and 251 (42 %) were females. Overall, median percent inhibition to BA.4/5 and WT was 71.61 % (IQR 39.44–92.82) and 98.60 % (IQR 83.07–99.73), respectively. Those with a past SARS-CoV-2 infection had a higher median percent inhibition to BA.4/5 and WT, when compared to uninfected individuals overall and when stratified by two or three vaccine doses. When comparing two vs. three WT vaccine doses among SARS-CoV-2 negative participants, we did not detect a difference in BA.4/5 percent inhibition, but there was a difference in WT percent inhibition. Among those with previous SARS-CoV-2 infection(s), when comparing two vs. three WT vaccine doses, there was no observed difference between groups. These findings demonstrate that additional Whttps://www.covid19immunitytaskforce.ca/citf-databank/#accessing https://www.covid19immunitytaskforce.ca/citf-databank/#accessinguhan Hu-1 platform mRNA vaccines did not improve NtAb response to BA.4/5, but prior SARS-CoV-2 infection enhances NtAb response.

## Data Summary

All supporting data can be found at: https://www.covid19immunitytaskforce.ca/citf-databank/#accessing

## Introduction

The severe acute respiratory syndrome coronavirus 2 (SARS-CoV-2), causing the coronavirus disease 2019 (COVID-19) pandemic, has resulted in millions of deaths worldwide [1]. The development and widespread distribution of mRNA COVID-19 vaccines, such as BNT162b2 and mRNA-1273, have minimized severe COVID-19 illness and death [2]. Previous studies have found robust immunological responses to wild-type and Delta lineages of SARS-CoV-2 at 6 months post-initial vaccination, particularly with two doses of mRNA-1273 [3]. However, continued evolution of the SARS-CoV-2 virus has introduced several novel variants since the initial doses of mRNA vaccine were administered. Thus, long-term follow-up of immunogenicity is warranted. The highly contagious BA.1 (Omicron) variant of concern was first detected in Canada in November 2021. Due to its increased transmissibility, Omicron led to a surge of new SARS-CoV-2 infections and became the predominant circulating lineage by 2022 [4]. Various subvariants of Omicron, including BA.4/5, subsequently emerged and continue to impact public health globally [5]. In response, several booster vaccination campaigns have been launched and offered additional doses of the original, wild-type (WT) Wuhan Hu-1 platform mRNA vaccines. However, it is unclear if additional wild-type mRNA vaccines have incremental benefit during the Omicron era.

As new SARS-CoV-2 lineages arise, there is less clarity regarding the long-term effectiveness and immunogenicity of the original mRNA vaccines against novel Omicron lineages. In addition, with a large magnitude of the population having been previously infected with SARS-CoV-2, particularly Omicron, it would also be beneficial to understand immunogenicity elicited by previous infection and whether booster wild-type vaccine doses confer additional protection. Such knowledge will also have implications for future waves of infection, during which the available vaccines do not match the circulating strains. For the above reasons, we sought to investigate the humoral immunogenicity against the WT and Omicron BA.4/5 strains at 18 months post-initial mRNA vaccine, comparing groups based on past SARS-CoV-2 infection and the number of WT-directed mRNA vaccine doses.

## Methods

### Study design and setting

Samples for this analysis were selected from participants in the ‘COVID-19 Occupational Risks, Seroprevalence, and Immunity among Paramedics in Canada (CORSIP)’ study, who were working paramedics in the Canadian provinces of British Columbia, Alberta, Saskatchewan, Manitoba, or Ontario. The CORSIP study began enrolling participants in January 2021, after receiving research ethics board approvals from the University of British Columbia (H20-03620) and the University of Toronto (40435). Participants provided electronic consent upon enrolment and completed questionnaires regarding health and sociodemographic information, COVID-19 vaccination history and status, and history of SARS-CoV-2 infections confirmed by positive polymerase chain reaction (PCR) test and/or rapid antigen test (RAT) results. Participants were asked to provide blood samples and survey data at 6 month intervals, including at an 18 month timepoint, following their first dose of a COVID-19 mRNA vaccine (if vaccinated).

### Participant and sample selection

Among CORSIP participants enrolled between January 2021 and November 2022, we included participants who had received two or three doses of any Health Canada approved mRNA COVID-19 vaccine (BNT162b2 and mRNA-1273). We focused on these vaccines, given that the vast majority of CORSIP participants were recipients. Participants were included if they had provided a blood sample at 18 months ±2 weeks from the date of their first vaccine dose. We excluded participants: (1) who received a bivalent vaccine (bivalent vaccines had just been released at the 18 month timepoint and very few of our participants had received them); (2) who only received one vaccine dose; (3) who received four vaccine doses; (4) who received non-mRNA vaccines; (5) who had incomplete vaccine and/or infection history (e.g. vaccine or infection date or type of vaccine missing); (6) who had a self-reported previous SARS-CoV-2 infection or COVID-19 vaccination within 60 days prior to this blood collection timepoint (given the expected immunological response in this initial phase post-antigen exposure).

### Laboratory testing

All samples were tested with the V-PLEX SARS-CoV-2 Panel 28 ACE2 Kit (Meso Scale Discovery, MD, USA) to measure the percent inhibition of ACE2 for both the wild-type Wuhan Hu-1 and BA.4/5 spike antigen. This assay platform has previously been shown to perform as a reliable surrogate for live virus neutralization [6, 7]. All blood serum samples were tested according to the manufacturer’s instructions. All samples were also tested with the Roche Elecsys Anti-SARS-CoV-2 Nucleocapsid (N) protein assay (Roche Diagnostics Corp., Indianapolis, IN, USA) assay to immunologically identify samples from participants with previous SARS-CoV-2 infection.

### Variable definitions

A past SARS-CoV-2 infection was defined as: (1) self-reported positive result on a rapid antigen test (RAT) or polymerase chain reaction (PCR) test; or, (2) a reactive Roche Elecsys Anti-SARS-CoV-2 N assay. We classified previous SARS-CoV-2 infections as Omicron vs. pre-Omicron, which was determined based on the date of the participant’s last self-reported positive PCR or RAT test result: a positive test result on 1 January 2022 or later were defined as having an Omicron infection, whereas those with positive results on 26 November 2021 or earlier were considered infected with a pre-Omicron lineage. The majority of COVID-19 cases in Canada beyond this date were Omicron [4]. We considered those with positive results between 27 November 2021 to 31 December 2021 to have an unspecified infection (and thus excluded from Omicron vs pre-Omicron comparisons) to account for a combination of pre-Omicron and Omicron lineages circulating during this period. For cases that were classified as having a previous SARS-CoV-2 infection based on a reactive Elecsys test, to control for the possibility of a pre-Omicron antibody being detected during the Omicron time period, SARS-CoV-2 infections identified by Roche Nucleocapsid assay had to have a reactive test during the Omicron period and a previous non-reactive test(s) to be considered an Omicron infection (otherwise these were excluded from Omicron vs pre-Omicron comparisons).

### Outcome measures

The primary and secondary outcomes were ACE2 percent inhibition to the BA.4/5 and WT antigens, respectively. Due to being the predominant circulating lineages at the time of blood collection [4], BA.4/5 lineages were specifically selected for analysis.

### Statistical analyses

Analyses were performed using GraphPad Prism Version 9.5.0 (GraphPad Software, San Diego, CA). Participant characteristics and outcomes were reported as counts (with percentages) for categorical variables, and median (with interquartile range [IQR]) for continuous variables. Outcome measures were reported as median with interquartile range (IQR). The median percent inhibition between two groups was compared using a non-parametric Mann-Whitney U test. A *P* value of less than 0.05 was considered statistically significant.

We performed several comparisons. First, we compared groups based on whether the participant had received two vs. three vaccines, to investigate the potential impact of repeated dosing with an ancestral strain vaccine on humoral response to an antigenically divergent, more contemporary variant (BA.4/5). Second, we divided participants into four groups, based on the number of vaccines and past SARS-CoV-2 infection history (two doses and no previous SARS-CoV-2 infections [‘two doses uninfected’], three doses and no previous SARS-CoV-2 infections, two doses and previous SARS-CoV-2 infection(s) [‘two doses infected’], and three doses and previous SARS-CoV-2 infection(s)) and compared each group to all others. In the third comparison, we further divided the subgroups with previous SARS-CoV-2 infection(s) into those who had a pre-Omicron vs. Omicron vs. unspecified infection, to investigate whether a prior Omicron infection elicited greater NtAb response.

## Results

**Fig. 1.**
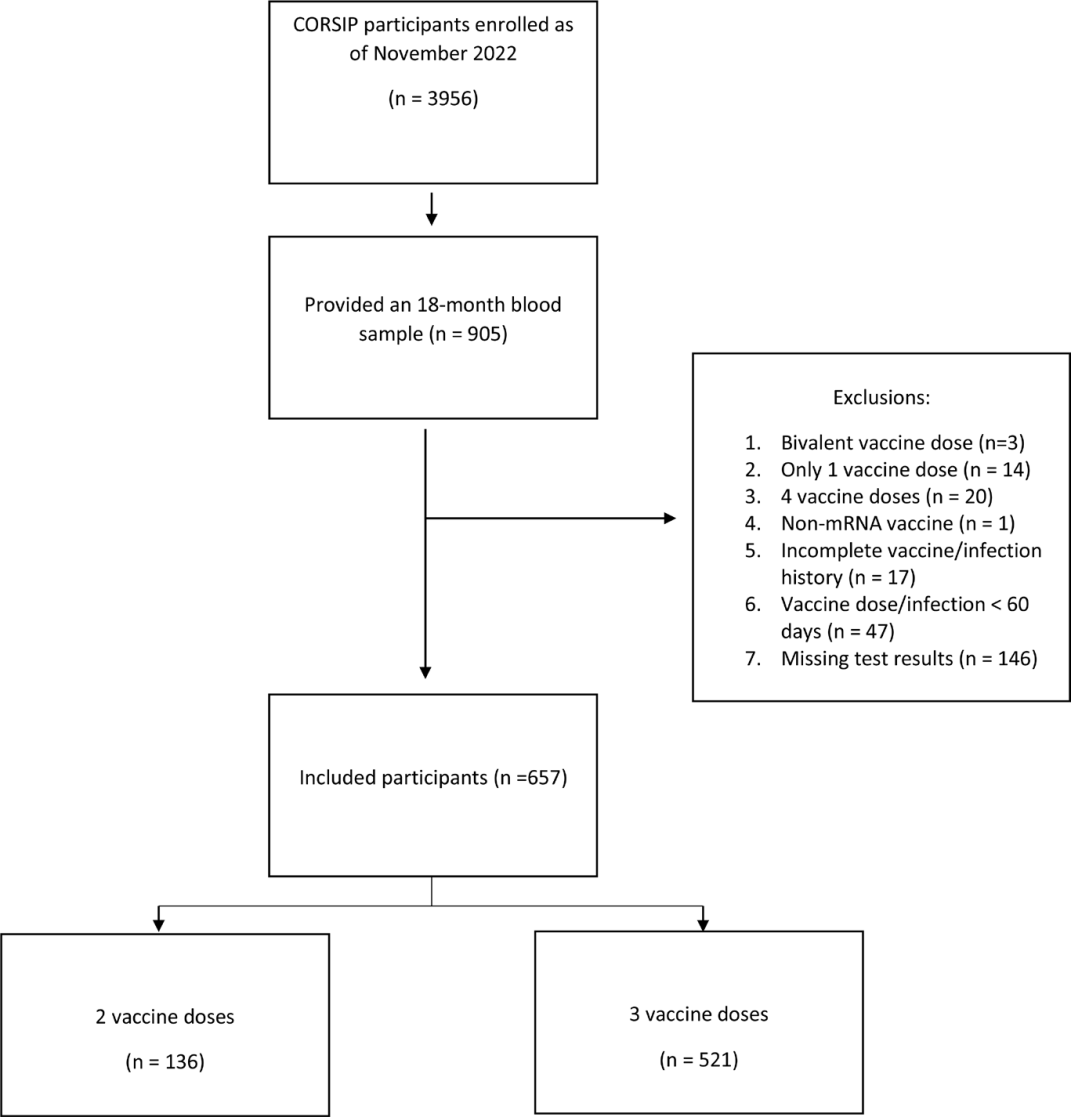
Participant selection flow diagram

This study included a total of 657 participants out of 3956 enrolled as of November 2022, 251 (42 %) of whom were female (Fig. 1). The median participant age was 40 years (IQR 33–50 years); 18 month blood samples were collected between June 2022 and November 2022. Participants had a median of 248 days (IQR 224–276 days) between their last vaccination date and their blood collection, and half of the participants were vaccinated exclusively with BNT162b2. Additional participant characteristics are summarized in Table 1.

**Table 1.** Participant characteristics at 18 month blood collection from first original mRNA vaccine date

Characteristics	Two vaccines (*n*=136)	Three vaccines (*n*=521)
**Age** (years), median (IQR)	38 (31–50)	40 (34–50)
**Sex**, n (%)		
Female	43 (39)	208 (40)
Male	67 (61)	285 (55)
Missing	26 (19)	28 (5.4)
**Last vaccine-to-BC interval** (days), median (IQR)	485 (431–507)	241 (217–255)
**Second vaccine to BC** (days), median (IQR)	485 (431–507)	501 (447–510)
**COVID+ history**, n (%)	94 (69)	277 (53)
Omicron COVID	71 (52)	210 (40)
Pre-omicron COVID	7 (5.1)	23 (4.4)
Unspecified COVID	16 (12)	44 (8.4)
**COVID-to-BC interval** (days), median (IQR)	160 (116–197)	145 (95–202)
Missing, N (%)	29 (21)*	96 (18)*
**ACE2 percent inhibition**, median (IQR)		
BA.4/BA.5	75 (40–94)	69 (39–93)
Wuhan Hu-1	99 (83–100)	99 (83–100)
**Vaccine 1**, n (%)		
mRNA-1273	33 (24)	157 (30)
BNT162b2	103 (76)	364 (70)
**Vaccine 2**, n (%)		
mRNA-1273	101 (74)	154 (30)
BNT162b2	35 (26)	367 (70)
**Vaccine 3**, n (%)		
mRNA-1273	–	256 (49)
BNT162b2	–	265 (51)

SD, standard deviation; COVID+, prior SARS-CoV-2 infection; COVID-, uninfected individual; Omicron COVID, SARS-CoV-2 infection reported on 1 January 2022 or later; Pre-omicron COVID, SARS-CoV-2 infection reported on 26 November or prior; unspecified infection reported between 27 November 2021 to 31 December 2021 or prior SARS-CoV-2 infection determined by reactive N-Roche assay with no prior unreactive N-Roche result; BC, blood collection

*, participants determined to be positive through N-Roche assay where date of COVID-19 is unknown.

**Fig. 2.**
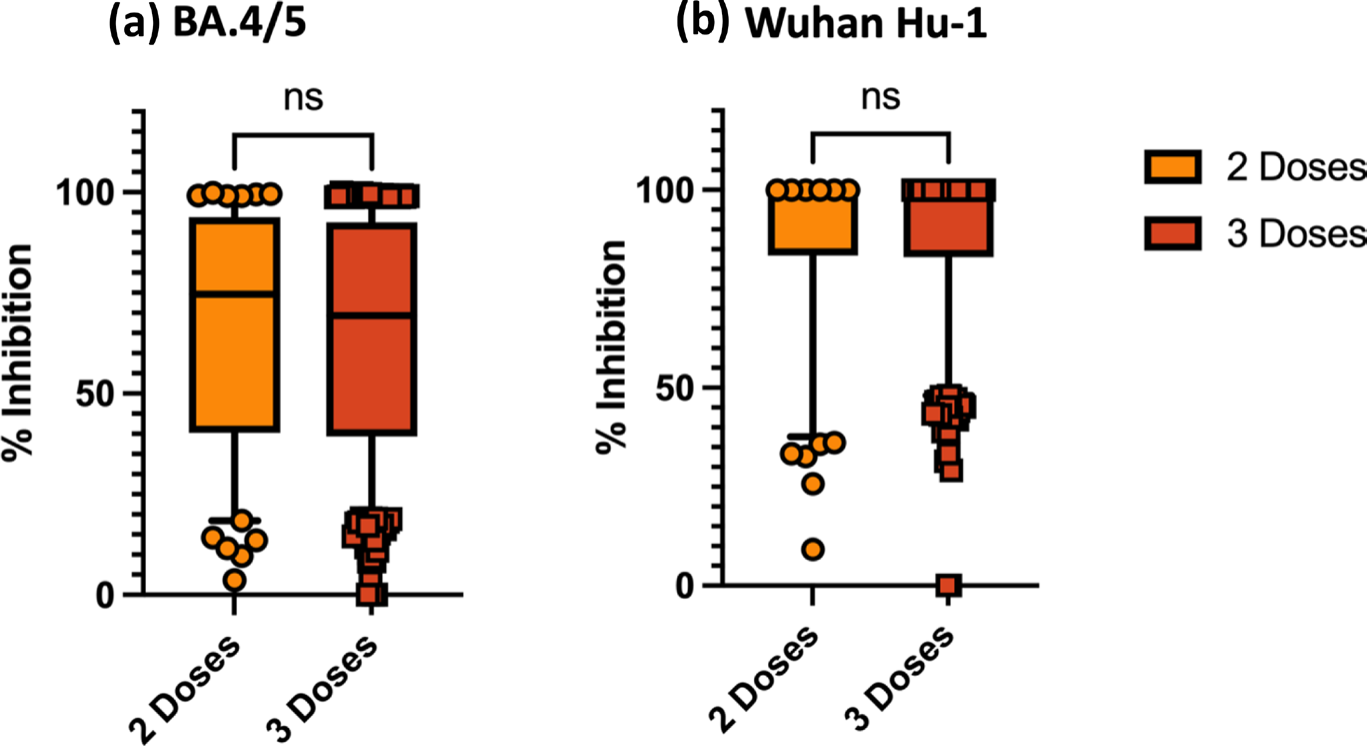
ACE2 percent inhibition to (**a**) BA.4/5 and (**b**) Wuhan Hu-1 by vaccine dose. ns, not significant; box plot shows median and interquartile range (IQR); whiskers, 5-95^th^ percentile; two doses, *n*=136; three doses, *n*=521.

In the first comparison of two vs. three vaccines (Fig. 2), we observed no significant difference in percent inhibition to BA.4/5 with two vaccine doses (*n*=136; 74.67%, IQR 40.24–93.82) vs. three vaccine doses (*n*=521; 69.30 %, IQR 39.34–92.60). Similar findings are observed in Fig. 2B when comparing two doses (*n*=136; 98.75 %, IQR 83.48–99.75) with three doses (*n*=521; 98.54 %, IQR 83.06–99.73) ACE2 percent inhibition to WT.

**Fig. 3.**
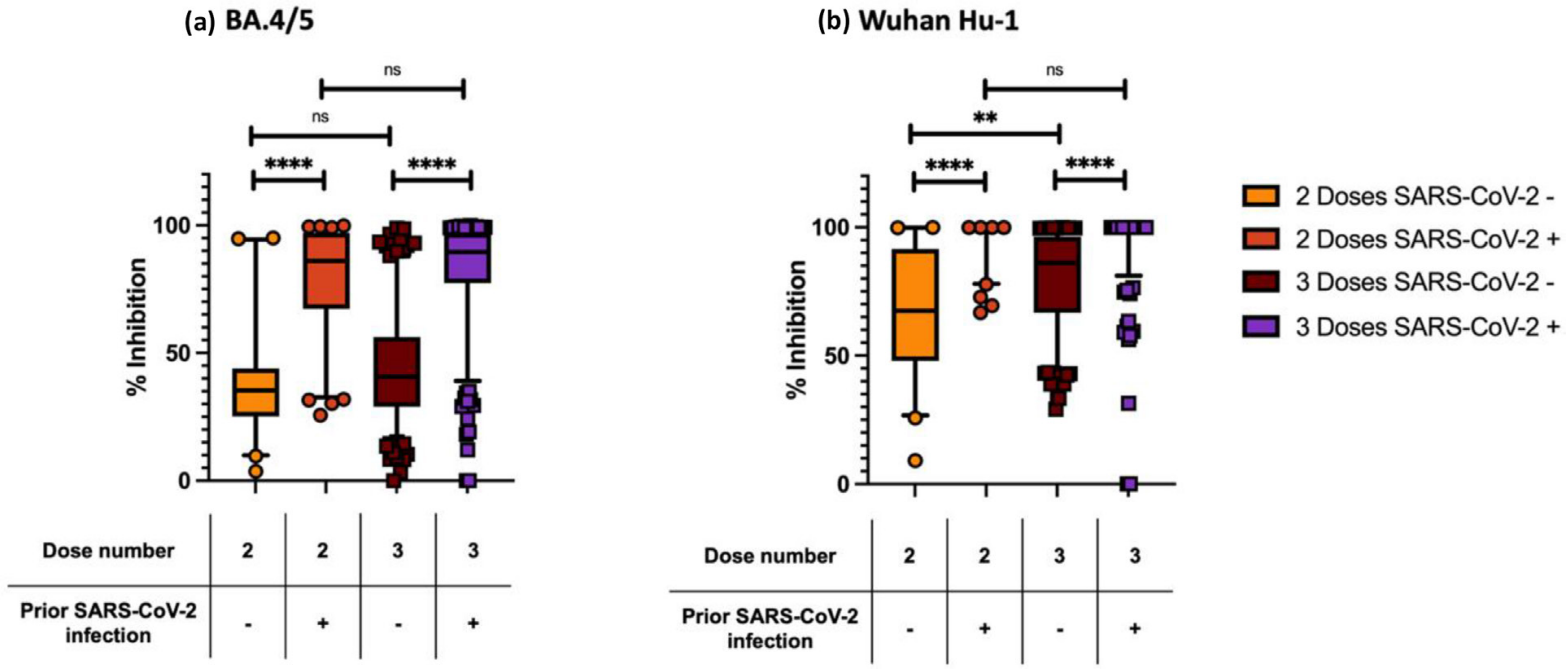
ACE2 percent inhibition to (**a**) BA.4/5 and (**b**) Wuhan Hu-1 by vaccine dose and prior SARS-CoV-2 infection status*. P*<0.0001, ****; *P*<0.0021,** ; ns, not significant; box plot shows median and interquartile range (IQR); whiskers, 5-95^th^ percentile; -, no prior SARS-CoV-2 infection; +, prior SARS-CoV-2 infection; two doses no prior infection, *n*=42; two doses with prior infection, *n*=94; three doses no prior infection, *n*=244; three doses with prior infection, *n*=277. Comparisons were performed between two groups at a time.

Fig. 3 shows the results of the second comparison where participants are divided into four subgroups based on the number of vaccines and SARS-CoV-2 infection history. For percent inhibition against BA.4/5, the median percent inhibition was: (1) 35.4 % (IQR 25–44) for two doses uninfected (*n*=42); (2) 86.0 % (IQR 67–97) for two doses infected (*n*=94); (3) 40.7 % (IQR 29–56) for three doses uninfected (*n*=244); and, (4) 89.5 % (IQR 77–97) for three doses infected (*n*=277). Within all four subgroups, those with a previous, unspecified SARS-CoV-2 infection(s) had a greater percent inhibition against both BA.4/5 and WT, when compared to uninfected participants. When examining BA.4/5, we did not detect a difference between those with two vs. three vaccines, when comparing amongst the infected or uninfected cases. When examining WT ACE2 inhibition, we observed a difference between those with two vs. three vaccine doses in uninfected participants, but not when examining previously infected participants.

**Fig. 4.**
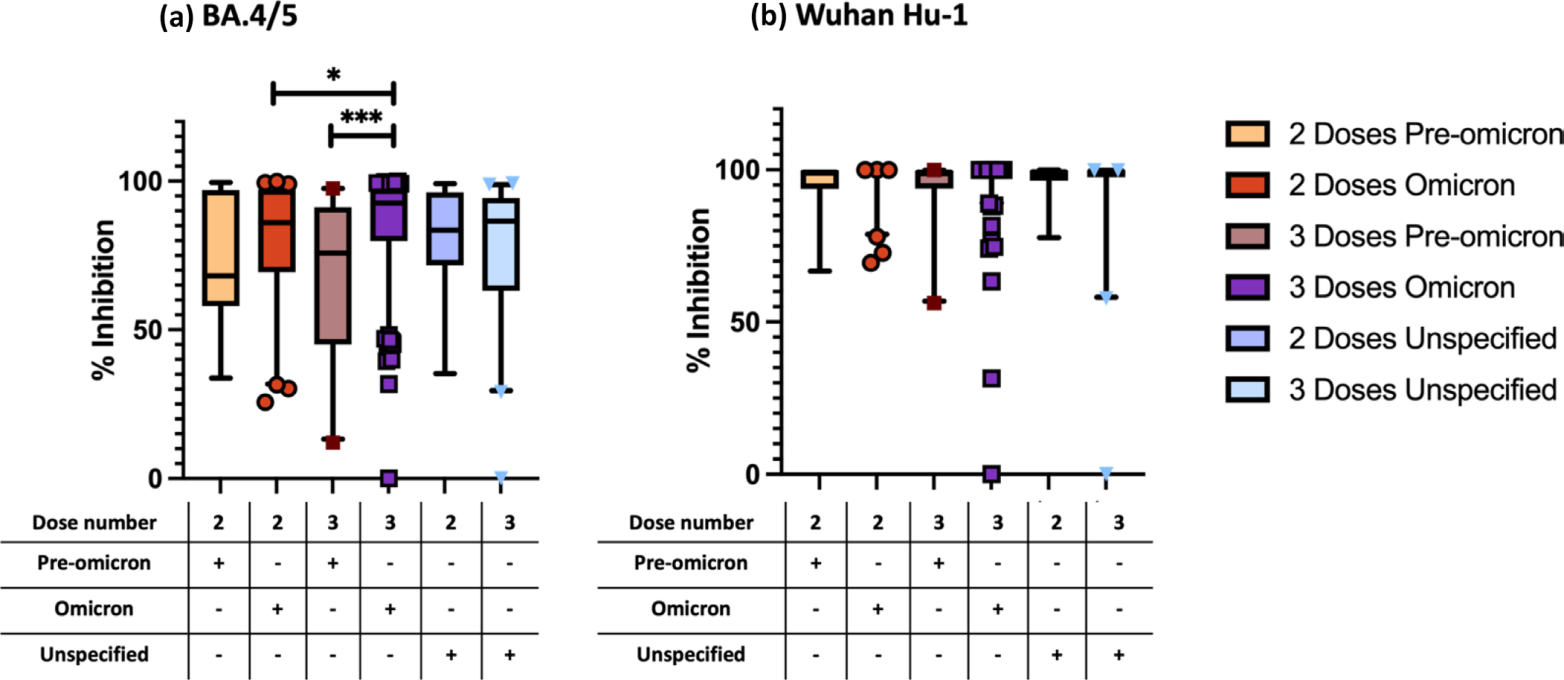
ACE2 percent inhibition to (A) BA.4/5 and (B) Wuhan Hu-1 by vaccine dose and pre-Omicron, Omicron, or unspecified SARS-CoV-2 infection status. P < 0.001, ***; P < 0.05, *; ns, not significant; box plot shows median and interquartile range (IQR); whiskers, 5-95th percentile; +, indicates a prior SARS-CoV-2 infection type; −, indicates the absence of a prior SARS-CoV-2 infection type; two doses prior pre-omicron infection, n = 7; two doses prior omicron infection, n = 71; two doses prior unspecified infection, n = 16; three doses prior pre-omicron infection, n = 23; three doses prior omicron infection, n = 210; three doses prior unspecified infection, n = 44. Comparisons were performed between two groups at a time.

Fig. 4 shows results of the third comparison based on the type of preceding SARS-CoV-2 infection strain. We observed significantly greater (*P*<0.001) percent inhibition to BA.4/5 when comparing individuals with three doses and Omicron infection (*n*=210; median 92.65, IQR [80-97]) with three doses and pre-Omicron infection (*n*=23; 75.83, IQR [45-91]) (Fig. 4). We also observed a significantly greater percent inhibition (*P*<0.05) in individuals with three doses and prior Omicron infection compared to those with two doses and prior Omicron infection. In contrast, percent inhibition to WT was consistently high across all groups and showed no significant differences with varying infection type or increasing number of vaccine doses (Fig. 4).

## Discussion

We examined NtAb response against the Omicron and wild-type strains from a prospective cohort of 657 Canadian paramedics. We observed that those with prior SARS-CoV-2 infection(s), compared to uninfected individuals, demonstrated greater ACE2 percent inhibition against WT and BA.4/5 lineages. Interestingly, our data indicate that additional WT-directed vaccines did not lead to either enhanced nor reduced humoral immunogenicity against more recent Omicron variants, regardless of whether they had been previously infected or not. These data suggest that providing additional WT-directed vaccine doses after two doses did not provide additional benefit in the current time period, which may have implications for future decisions regarding additional dosing strategies when available vaccines do not match the current circulating strains.

Overall, an increased number of WT mRNA vaccine doses was not associated with an increased percent inhibition against BA.4/5, except in those individuals with two doses and a prior Omicron era infection. These findings differ from prior studies that examined antibody neutralization against wild-type [8] and original Omicron (B.1.1.529) variants [9] with two doses showing reduced NtAb response. However, these studies only assessed responses after relatively short periods post-second doses (up to 7 months). Several studies have shown greater NtAb responses among vaccinated individuals with a prior infection compared to those without [10, 11]. However, two studies have found no difference due to prior BA.1 infection between two vaccination and three vaccination groups [12, 13]. Carazo *et al.* found no significant difference in reducing the risk of infection to BA.2 from a prior BA.1 infection across two vaccine doses vs. three vaccine doses [12], and Zheng *et al.* found no difference in 50 % neutralization titre (NT50) in prior BA.1 infected individuals with either two or three vaccine doses [13]. When compared to our findings, the observed differences could be attributed to methodological differences such as the intervals between sample testing and prior infection(s), different SARS-CoV-2 variants, COVID-19 vaccine types and outcome measures selected, and study design used. Further, the statistically significant improvement in percent inhibition in those with prior Omicron infection and a third dose observed in our data could also be due to differences in subgroup sample sizes and characteristics such as vaccine type (e.g. BNT162b2 and mRNA-1273), vaccination intervals, and the possibility of multiple prior infections, which were not able to be estimated based on our study’s cohort. Although statistically significant, the median percent inhibition was relatively high across both groups and may not be clinically significant in the context of hospitalization rate, infection burden, and/or disease severity. The finding that additional Wuhan Hu-1 platform mRNA vaccines did not improve immunogenicity may have policy implications, given that use of these vaccines may have little further utility, when given alone or in combination with vaccines directed at other strains.

A potential concern with repeated vaccine doses using antigen from an ancestral strain is the phenomenon of ‘original antigenic sin’, where repeated exposure to one antigen may result in the immune response being preferentially directed towards the primary antigen even when infected with a new variant/strain. This has been described with influenza and other RNA viruses [14, 15]. Interestingly, our data shows that additional vaccine doses against the wild-type SARS-CoV-2 strain did not appear to diminish the median percent inhibition against BA.4/5. This could be due to the mRNA vaccines eliciting some cross-neutralization against variants of concern (VoC), such as Omicron and its sublineages [16]. However, these vaccinated individuals may still potentially be susceptible to VoCs, given the observed differences in median percent inhibition to BA.4/5 and WT, regardless of the number of vaccine doses or prior SARS-CoV-2 infection. Thus, our findings contribute to the current literature in supporting vaccine guidelines that emphasize the importance of bivalent vaccines designed to target Omicron variants, rather than providing boosters against the original strain [17, 18]. As new SARS-CoV-2 lineages emerge and new vaccines are designed, our findings also provide some insight into biological patterns of immune response related to prior infection and vaccination dose.

This study has limitations. Firstly, we used percent ACE2 inhibition for the outcome measure, which is a surrogate marker for immunity. Although not as clinically relevant as clinical outcomes, ACE2 inhibition has been shown to correlate with live virus neutralization (the gold standard for antibody efficacy and predictive of clinical immune response [19–21], and has been used extensively as a marker for immunity in other studies [3, 6, 7, 22, 23]). We utilized self-reported data regarding participant characteristics, which are prone to recall bias, inaccuracies, and incompleteness. The Roche Elecsys Anti-SARS-CoV-2 N assay used to classify some self-reported SARS-CoV-2 negative participants as SARS-CoV-2 positive is reported to have a 90 % sensitivity [24]. Further, these positive participants were missing the date of their prior infection. Additionally, we were unable to determine if participants had multiple prior infections or completely estimate the time interval from infection to blood draw due to the study design and unreliability of self-reported data. No post-hoc correction was applied in our statistical analysis. Due to the observational nature of this study, comparisons were made between potentially uneven groups that may differ in measured and/or unmeasured characteristics. For example, one such confounder would be the lack of participant data on potential therapies or medications taken that could alter the immune response such as corticosteroids or chemotherapy. Lastly, the lack of data on participant comorbidities could impact immune response strength [25].

## Conclusion

Those with previous SARS-CoV-2 infection demonstrated higher ACE2 percent inhibition against WT and BA.4/5 antigens, compared to those without a prior infection. Three vs. two Wuhan Hu-1 platform vaccines doses improved percent inhibition to WT, but not BA.4/5 antigens.
